# A multimodal machine learning model for predicting dementia conversion in Alzheimer’s disease

**DOI:** 10.1038/s41598-024-60134-2

**Published:** 2024-05-29

**Authors:** Min-Woo Lee, Hye Weon Kim, Yeong Sim Choe, Hyeon Sik Yang, Jiyeon Lee, Hyunji Lee, Jung Hyeon Yong, Donghyeon Kim, Minho Lee, Dong Woo Kang, So Yeon Jeon, Sang Joon Son, Young-Min Lee, Hyug-Gi Kim, Regina E. Y. Kim, Hyun Kook Lim

**Affiliations:** 1 Research Institute, Neurophet Inc., Seoul, 06234 Republic of Korea; 2 Department of Psychiatry, Seoul St. Mary’s Hospital, College of Medicine, The Catholic University of Korea, Seoul, 06591 Republic of Korea; 3 Department of Psychiatry, Chungnam National University Hospital, Daejeon, 35015 Republic of Korea; 4 Department of Psychiatry, College of Medicine, Chungnam National University, Daejeon, 35015 Republic of Korea; 5 Department of Psychiatry, Ajou University School of Medicine, Suwon, 16499 Republic of Korea; 6 Department of Psychiatry, Pusan National University School of Medicine, Pusan National University, Busan, 49241 Republic of Korea; 7 Department of Radiology, Kyung Hee University Hospital, Kyung Hee University School of Medicine, Seoul, 02447 Republic of Korea; 8 Department of Psychiatry, Yeouido St. Mary’s Hospital, College of Medicine, The Catholic University of Korea, 10 63-ro, Yeongdeungpo-gu, Seoul, 07345 Korea; 9 CMC Institute for Basic Medical Science, the Catholic Medical Center of The Catholic University of Korea, 222 Banpo-daero, Seocho-gu, Seoul, 06591 Republic of Korea

**Keywords:** Neuroscience, Medical research, Neurology, Nanoscience and technology

## Abstract

Alzheimer’s disease (AD) accounts for 60–70% of the population with dementia. Mild cognitive impairment (MCI) is a diagnostic entity defined as an intermediate stage between subjective cognitive decline and dementia, and about 10–15% of people annually convert to AD. We aimed to investigate the most robust model and modality combination by combining multi-modality image features based on demographic characteristics in six machine learning models. A total of 196 subjects were enrolled from four hospitals and the Alzheimer’s Disease Neuroimaging Initiative dataset. During the four-year follow-up period, 47 (24%) patients progressed from MCI to AD. Volumes of the regions of interest, white matter hyperintensity, and regional Standardized Uptake Value Ratio (SUVR) were analyzed using T1, T2-weighted-Fluid-Attenuated Inversion Recovery (T2-FLAIR) MRIs, and amyloid PET (αPET), along with automatically provided hippocampal occupancy scores (HOC) and Fazekas scales. As a result of testing the robustness of the model, the GBM model was the most stable, and in modality combination, model performance was further improved in the absence of T2-FLAIR image features. Our study predicts the probability of AD conversion in MCI patients, which is expected to be useful information for clinician’s early diagnosis and treatment plan design.

## Introduction

Alzheimer’s disease (AD) is the most common neurodegenerative disorder, accounting for 60–70% of patients with dementia^1^. Throughout the course of neurodegeneration, cognitive function and daily functional abilities deteriorate progressively. Mild cognitive impairment (MCI) is a diagnostic entity defined as an intermediate stage between subjective cognitive decline and dementia^2^. Among patients with MCI, the rate of conversion to dementia is known to be around 10–15% annually^3–5^. AD, a representative of these degenerative disease, is characterized by extensive synapse loss and neuronal death (atrophy) within the brain, as well as the formation of intracellular neurofibrillary tangles and extracellular β-amyloid plaques^6^. The neuropathological progression of AD may be detected as an MCI-like status for years before clinical symptoms become evident^7^. For this reason, there is a need to detect and prevent AD earlier through clinically detectable MCI during AD progression. Among different neuroimaging modalities, magnetic resonance imaging (MRI) and positron emission tomography (PET), technologies for analyzing brain patterns and the underlying pathologies of AD are widely used in AD-related research^8^. Over the years, much research has been conducted to discover meaningful biomarkers that may be useful in predicting AD conversion in MCI patients from neuroimaging. There are previous studies to find predictors of AD conversion in MCI patients from T1-weighted images^8–14^. Moradi et al. reported that the accuracy of classifying stable MCI and progressive MCI patients was improved when aggregating not only region of interest (ROI) volume information but also age and cognitive measures^8^. Misra et al., Karas, G., et al., Risacher, Shannon L., et al. reported that the volume of gray matter regions and white matter of the converters was significantly smaller than the non-converters, and the overall brain atrophy pattern in the conversion group was like that in AD patients^9,12,13^. There is also a previous study that created a model to classify between MCI patients who converted to AD and MCI patients who did not convert to AD using not only the volume of T1-weighted images, but also 3D texture, ApoE ε4 genotype, and cognitive test score^10^. To predict AD conversion in MCI patients, voxel-based features of T1-weighted images were extracted based on voxel-based morphometry, hippocampus volumes, volumes of the entorhinal cortex, and a set of regional volumetric, surface area, and cortical thickness measures across the brain. When applied to a machine learning model, performance was good when hippocampus volume, entorhinal cortex volume, and regional volumetric were used^11^. There is also a report that using hippocampus grade as a feature of another T1-weighted image, the accuracy increased more when hippocampus grade was used as a predictor of AD conversion in MCI patients than when hippocampus volume was used^14^. Hippocampal occupancy (HOC), an indicator of cerebral atrophy due to degenerative brain disease, was added as a feature of T1-weighted images. HOC was calculated as the volume ratio of the hippocampus region to the inferior lateral ventricle^15^. In this study, the features of T1-weighted images used included not only regional brain volume but also HOC. On the other hand, according to research on the relationship between white matter hyperintensity (WMH) in T2-FLAIR image and Alzheimer’s disease, it is suggested that WMH can have independent effects on cognitive function, neuropsychiatric symptoms, and functional decline related to the progression of Alzheimer’s disease^16^. According to a study comparing the regression of WMH with brain atrophy and changes in cognitive profiles, it has been shown that when WMH regress, the extent of brain atrophy decreases, and cognitive function improves. Additionally, significant differences were reported in the improvement of cognitive function between the regressed group and the stable group with WMH^17^. In this study, the features of T2-FLAIR imaging included WMH, which have an independent effect on cognitive function, and these were evaluated using the Fazekas scale, rating with 0–3^18^. Meanwhile, according to a long-term study investigating β-amyloid deposition and the progression of dementia in MCI due to AD using amyloid positron emission tomography (αPET) imaging, it was reported that β-amyloid deposition increases over time and that the degree of deposition is associated with an increased risk of conversion to AD^19^. Additionally, in a study utilizing a machine learning algorithm-based classification model for early diagnosis of dementia in patients with MCI, it was shown that features extracted from αPET images are effective in predicting early diagnosis of AD and the conversion of patients with MCI^20^. The results demonstrated that the application of the SUVR (Standardized Uptake Value Ratio) values, which quantifies β-amyloid deposition in αPET images, in the prediction model for AD progression, showed its utility in predicting the progression of AD^21,22^.

Based on the evidence that these features from each imaging modality contribute to the prediction of AD conversion, numerous studies using machine learning for AD conversion prediction have been conducted. Cheng, Bo, et al., reported that by combining MRI, FDG-PET, and cerebrospinal fluid (CSF) biomarker features and applying manifold-regularized transfer learning, they improved the performance of the AD conversion prediction model for MCI patients up to an accuracy of 80%^23^. Rana, Sijan S., et al., trained a conversion prediction model using deep learning with T1-weighted images, age, sex, apolipoprotein ε4 carriers (ApoE4), and neurophysiological test scores. The accuracy of the model was reported to be 69.8%^24^. Minhas, Sidra et al., combined MRI images and neurophysiological measures to perform longitudinal trajectory modeling for the early prediction of AD conversion in MCI patients. The results showed that this combined approach was useful for predicting conversion at an early stage, and it also aided in early diagnosis and personalized treatment planning^25^. Li, Hai-Tao et al. enhanced the predictive ability of AD conversion risk in MCI patients by considering the differences in progression rate, clinical characteristics, and treatment response among MCI patients and stratifying them based on their genetic and molecular characteristics^26^.

Many studies have been conducted to predict the conversion from MCI to AD by combining MRI images and neurophysiological test scores. Previous research has shown that the image features from MRI and PET images can also provide valuable insights for predicting AD conversion. However, there is a scarcity of research on the combination of multiple modalities and the selection of the most suitable models for this purpose. Therefore, in this study, we aim to explore the machine learning model algorithm and modality combination that are suitable for the AD conversion prediction model by incorporating not only well-known features such as regional volume, HOC, WMH, Fazekas scale, and regional SUVR but also additional factors including age, sex, mini-mental state examination (MMSE), ApoE4.

## Methods

### Data acquisition

A total of 196 subjects were enrolled from four tertiary hospitals and the Alzheimer’s Disease Neuroimaging Initiative (ADNI) dataset. Within a four-year follow-up period, we defined the subjects as the AD conversion group when their global Clinical Dementia Rating (CDR) score reached 1.0 or higher within the follow-up period. Subjects maintaining a global CDR score of 0.5 were defined as the non-conversion group. The collected demographics of all sites are (1) age, (2) sex, (3) MMSE, (4) ApoE4, (5) CDR. Those aged 50-85 years, diagnosed with MCI at the time of initial treatment, and who underwent follow-up diagnostic tests within 2–4 years were included in the eligibility criteria. Approval of the MRI and *α*PET images used for this study was obtained from the Yeouido St. Mary’s Hospital Institutional Review Board (IRB) [2022-1185], the IRB of Chungnam National University Hospital (CNUH-2022-05-020), the IRB of Ajou University Hospital (AJIRB-MED-EXP-22-284) and the IRB of Kyung Hee University Hospital (KNUH-2022-05-012) with a waiver of informed consent. All conformed to the Declaration of Helsinki (https://www.nature.com/srep/journal-policies/editorial-policies#experimental-subjects). Image acquisition methods are described for each site. Site1 dataset underwent to brain MRI and PET at the Catholic University of Korea, Yeouido St. Mary’s Hospital, Seoul, Republic of Korea. A dataset satisfying the conversion definition was extracted and 44 non-conversion groups were obtained. MRI and PET images were obtained from patients with mild cognitive impairment. The site1 dataset was acquired from human subjects on 3.0T a Siemens scanner. T1-weighted MRI images were acquired (TR=1700~1800ms, TE=2.6ms, and flip angle=9°). T2 FLAIR MRI images were acquired (TR/TI=9000/2500ms, TE=76ms, Flip angle=150°). *α*PET images were acquired with ^18^F-Florbetaben, ^18^F-Flutemetamol.

The site2 dataset underwent brain MRI and PET at Chungnam National University Hospital, Daejeon, Republic of Korea. A dataset satisfying the conversion definition was extracted, and two non-conversion groups were obtained. MRI and PET images were obtained from patients with mild cognitive impairment. 3D T1-weighted MRI images were acquired on a 3.0T Siemens (TR=2000ms, TE=2.29ms, flip angle=8°), 3.0T GE (TR=7.956ms, TE=2.82ms, flip angle=10°). T2 FLAIR MRI images were acquired on a 3.0T Siemens (TR/TI=9000/2500ms, TE=121ms, Flip angle=121°), 3.0T GE (TR/TI=11000/2648.61ms, TE=93.544, flip angle=160°). *α*PET images were acquired with ^18^F-Flutemetamol.

The site3 dataset underwent to brain MRI and PET at the Ajou University Hospital, Suwon, Republic of Korea. A dataset satisfying the conversion definition was extracted, and 34 non-conversion and 3 conversion groups were obtained. MRI and PET images were obtained from patients with mild cognitive impairment. 3D T1-weighted MRI images were acquired on a 3.0 T GE (TR = 7.1 ~ 8.88 ms, TE = 2.776 ~ 3.396 ms, Flip angle = 8° or 12°), 3.0 T Philips (TR = 9.8 ms, TE = 4.6 ms, Flip angle = 8°). T2 FLAIR MRI images were acquired on a 3.0 T GE (TR/TI = 8800 ~ 12,000/2450 ~ 2709 ms, TE = 89 ~ 128 ms, Flip angle = 160°), 3.0 T Philips (TR/TI = 8000/2500 ms, TE = 125 ms, Flip angle = 90°). αPET images were acquired with 18F-Flutemetamol.

The site4 dataset underwent to brain MRI and PET at Kyung Hee University Medical Center, Seoul, Republic of Korea. A dataset satisfying the conversion definition was extracted, and 29 non-conversion and 14 conversion groups were obtained. MRI and PET images were obtained from patients with mild cognitive impairment. 3D T1-weighted MRI images were acquired on a 3.0 T Philips (TR = 9.4 ms, TE = 4.6 ms, Flip angle = 8°), 3.0 T Siemens (TR = 2000 ms, TE = 3.05 ms, Flip angle = 9°). T2 FLAIR MRI images were acquired using a 3.0 T Philips (TR/TI = 10,000/2800, TE = 120 or 125 ms, Flip angle = 90°) a 3.0 T Siemens (TR/TI = 8000 ~ 10,730/2500 ~ 2665.9 ms, TE = 86 ~ 115 ms, Flip angle = 150°). αPET images were acquired with 18F-Florbetaben.

For this study, we used the ADNIMERGE subset, in which demographic and clinical test scores and MRI and PET variables were summarized. This subset is part of the official dataset provided by the ADNI. When data satisfying the conversion definition were extracted from the subset, 40 non-conversion and 12 conversion groups were obtained. 3D T1-weighted MRI images were acquired on a 3.0 T GE (TR = 7.3 ~ 7.6 ms, TE = 3.05 ~ 0.12 ms, Flip angle = 11°), 3.0 T Philips (TR = 6.5 ms, TE = 2.9 ms, Flip angle:9°), 3.0 T Siemens (TR = 2300 ms, TE = 2.95 ~ 2.98 ms, Flip angle = 9°). T2 FLAIR MRI images were acquired on a 3.0 T GE (TR/TI = 4800/1442 ~ 1482 ms, TE = 115.7 ~ 117 ms, Flip angle = 90°), 3.0 T Philips (TR/TI = 4800/1650 ms, TE = 271 ~ 275 ms, Flip angle = 90°), 3.0 T Siemens (TR/TI = 4800 or 9000/1650 ~ 2500 ms, TE = 90 ~ 443 ms, 120°). αPET images were acquired using 18F-Florbetapir, 18F-Florbetaben.

### Image processing and image features preprocessing

The acquired 3D T1-weighted images and T2-FLAIR images were preprocessed and segmented into whole brain ROI regions^27^ and WMH regions using Neurophet AQUA (version 2.0, Neurophet Inc., Seoul, South Korea), a commercially available AI-based brain MRI analysis software^28^. After calculating the volume of the segmented area, intracranial volume (ICV) normalization was performed. The purpose of ICV normalization was to correct for differences in the ROI volume due to the different head sizes of individual and sexes. This was performed by dividing the total ICV by each volumetric feature of the subject. This normalization method is commonly used^29^. In addition, HOC, which is used as an index of neurodegenerative disease biomarkers^30^, was calculated and used as an input. WMHs ratio compared to white matter, periventricular WMHs ratio compared to white matter, and deep WMHs ratio compared to white matter were calculated, and the Fazekas scale was rated for each region as minimal (0), moderate (1), and severe (2) through segmented WMH regions of T2-FLAIR image^31^. The acquired *α*PET images were also registered with 3D T1-weighted images, the voxels in *α*PET images were scaled using the mean uptake value in the cerebellar gray matter to calculate the SUVR values using Neurophet SCALE PET (version 1.0, Neurophet, Seoul, South Korea). Consequently, 115 volumetric features were extracted from the T1-weighted images, 6 features from the T2-FLAIR images, and 144 regional SUVR values from the *α*PET images were used as each modality feature.

We split the dementia conversion group and non-conversion group so that they were composed of a certain ratio in the train set and test set. Since the ratio of the non-conversion group and the conversion group was about 4:1, we used the synthetic minority oversampling technique (SMOTE) to remove the possibility of biased prediction by balancing dementia conversion and non-conversion data. Before using data in a machine learning model, to ensure the same level of importance, standardization was performed on the train set and equally applied to the test set. Standardization was performed to ensure the same level of importance, and all features were used in the model. For this reason, the z score method was used, *z*_*j*_ = (*x*_*j*_-*µ*_*j*_) / *σ*_*j*_ where *x*_*j*_ is the original value for feature *j*, *z*_*j*_ is the normalized value, *µ*_*j*_ is the feature’s mean and *σ*_*j*_ is the feature’s standard deviation. Consequently, the *z*-score method produces a new dataset in which all features have zero mean and unit standard deviation. The values for categorical features were also encoded.

### Model selection

For the model selection, six widely used machine learning techniques were examined using training and testing set. The 196 dataset was divided in a stratified way into a training set (80%) and a testing set (20%), maintaining the sample percentage of each class in both sets. There were 100 pairs of training and testing sets were created for the preliminary test to investigate the robustness of the model. In each iteration, we trained each model and set up a grid search using the hyperparameters to select a model that generalized well. In the process of hyperparameters tuning, a 10-fold cross-validation was performed. The models used were decision trees (DT), random forests (RF), support vector machines (SVM), linear regression classifiers (LR), gradient boosting models (GBM), and Extreme Gradient Boosting (XGB). The testing set was applied to the tuned model to check the AUC distribution, and the model that showed the best robustness against data shuffling was finally selected. At this time, the standard for robustness was that mean AUC was high, the standard deviation AUC was small.

### Selection of modality combinations

For the selection of modality combination, the model training and performance evaluation were also conducted using 100 pairs of shuffled training set and testing set, like the model selection process. The model performance was investigated using a total of 11 modality combinations as follows: (1) demo (demographic characteristics), (2) A (*α*PET image features), (3) N (T1-weighted image features), (4) V (T2-FLAIR image features), (5) demo + A, (6) demo + N, (7) demo + V, (8) demo + AN, (9) demo + NV, (10) demo + AV, (11) demo + ANV. The AUC was calculated for each modality combination using the trained model, and a comparative analysis was performed to determine whether there were significant differences among the top three modality combination models and the demographic characteristics model. For the comparative analysis, the ensemble model of the shuffled testing set results for each subject was utilized.

### Performance estimates of final model

Based on the results of the model selection and the selection of modality combination, we estimated the performance of the final model using fixed training and testing set. In this process, we performed tenfold cross-validation for hyperparameter tuning. The trained model was then applied to the testing set, and the model performance was explored in terms of sensitivity, specificity, balanced accuracy, and area under ROC curve. (Eqs. 1, 2, 3, and 4):

1 $$Sensitivity\, \left( {SE} \right) = \frac{TP}{{{\text{TP}} + {\text{FN}}}}$$

2 $$Specificity\, \left( {SP} \right) = \frac{TN}{{{\text{TN}} + {\text{FP}}}}$$

3 $$Balanced\, Accuracy\, \left( {BA} \right) = \frac{{\left( {Sensitivity + Specificity} \right)}}{2}$$

4 $$AUC = Area\, under\, ROC\, curve$$

### Statistical analysis

Age, MMSE, and study interval information between the conversion group and the non-conversion group were compared and analyzed using a two-sample t-test. Chi-square tests were performed for sex and ApoE ε4 carriers. To compare the AUC of the model based on different modality combinations during the preliminary test, a DeLong test^32^ at the statistical significance level of 0.05.

## Results

### Patient’s demographics

The demographic characteristics of the participants are presented in Table 1. There was no statistically significant difference between the age (*t* = 1.08, *p* = 0.28), sex ratio (${\chi }^{2}$=0.04, *p* = 0.83), and study interval (*t* = 0.97, *p* = 0.34) of the conversion group and the non-conversion group. There was a statistically significant difference in ApoE4 ε4 carrier status (${\chi }^{2}$=5.11, *p* = 0.02) and MMSE (*t* = -4.68, *p* < 0.05). Participants with baseline CDR = 0.5 categorized into two groups of non-conversion group who maintained CDR score at 0.5 and conversion group who increased CDR score during follow up period. Detailed subject information for each site is shown in Supplementary Table 1, and scan parameters for each site for image acquisition are shown in Supplementary Table 2.

**Table 1:** Demographic characteristics of sample population.

Demographics	Non-converter 76% (149)	Converter 24% (47)	Statistic	*p*-value
Age (years), mean ± SD	71.66 ± 7.16	72.94 ± 6.64	1.08	0.28
MMSE, mean ± SD	26.46 ± 3.09	24.0 ± 3.24	-4.68	< 0.001
Female sex, % (N)	57 (85)	55 (26)	${\chi }^{2}$= 0.04	0.83
CDR (Baseline), mean ± SD	0.5	0.5	-	-
CDR (Follow up), mean ± SD	0.5	1.13 ± 0.33	-	-
ApoE ε4 carrier, % (N)	31 (46)	49 (23)	${\chi }^{2}$= 5.11	0.02
Study interval, years	2.61 ± 0.50	2.69 ± 0.54	0.97	0.34

SD, standard deviation; MMSE; Mini-mental State Examination, CDR; Clinical Dementia Rating. Chi-square tests for sex and ApoE ε4 carrier, two-sample t-tests for age, MMSE, and study interval.

### Model selection

The test results performed for model selection are described in Table 2. Table 3 shows the results of applying the testing set obtained by shuffling the tuned model for each modality combination. The highest mean AUC for each model was 0.728 for the demo + ANV combination in the DT model, 0.844 for the demo + AN combination in the RF model, 0.826 for the demo + AN combination in the SVM model, 0.809 for the demo + AV combination in the LR model, 0.881 for the demo + AN combination in the GBM model, and 0.865 for the demo + AN combination in the XGB model. As can be seen from the results, AUC generally tended to improve when a modality combination was used rather than using demographic characteristics or image features alone. As a result of examining the model’s robustness in terms of standard deviation of AUC, the model that showed the highest mean AUC and the smallest standard deviation was the GBM model.

**Table 2:** The AUC results of tenfold cross-validation of the training set obtained through 100 iterations of data shuffling.

Modality combination	Machine learning models					
	DT	RF	SVM	LR	GBM	XGB
	Mean ± SD	Mean ± SD	Mean ± SD	Mean ± SD	Mean ± SD	Mean ± SD
demo	0.772 ± 0.032	0.811 ± 0.029	0.792 ± 0.027	0.771 ± 0.023	0.796 ± 0.031	0.798 ± 0.027
A	0.827 ± 0.026	0.921 ± 0.015	0.952 ± 0.018	0.936 ± 0.017	0.918 ± 0.017	0.921 ± 0.014
N	0.822 ± 0.030	0.966 ± 0.008	0.991 ± 0.005	0.893 ± 0.026	0.967 ± 0.009	0.959 ± 0.010
V	0.682 ± 0.039	0.739 ± 0.032	0.688 ± 0.073	0.716 ± 0.040	0.720 ± 0.041	0.728 ± 0.040
demo + A	0.852 ± 0.025	0.938 ± 0.012	0.965 ± 0.012	0.944 ± 0.016	0.941 ± 0.013	0.944 ± 0.011
demo + N	0.837 ± 0.031	0.970 ± 0.008	0.991 ± 0.005	0.898 ± 0.025	0.968 ± 0.010	0.959 ± 0.011
demo + V	0.775 ± 0.035	0.853 ± 0.023	0.835 ± 0.030	0.811 ± 0.026	0.835 ± 0.030	0.842 ± 0.028
demo + AN	0.869 ± 0.027	0.967 ± 0.008	0.989 ± 0.005	0.957 ± 0.016	0.978 ± 0.009	0.977 ± 0.008
demo + NV	0.836 ± 0.030	0.969 ± 0.008	0.991 ± 0.005	0.906 ± 0.025	0.968 ± 0.009	0.960 ± 0.010
demo + AV	0.849 ± 0.027	0.941 ± 0.012	0.961 ± 0.014	0.945 ± 0.014	0.942 ± 0.012	0.946 ± 0.011
demo + ANV	0.871 ± 0.027	0.968 ± 0.008	0.989 ± 0.005	0.959 ± 0.014	0.978 ± 0.008	0.976 ± 0.007

AUC; Area Under ROC Curve, SD; Standard Deviation, DT; Decision Trees, RF; Random Forests, SVM; Support Vector Machines, LR; Linear Regression Classifiers, GBM; Gradient Boosting Models, XGB; Extreme Gradient Boosting, demo; demographic characteristics, A; amyloid PET image features, N; T1-weigted image features, V; T2-FLAIR image features.

**Table 3:** The AUC results of the testing set obtained through 100 iterations of data shuffling.

Modality combination	Machine learning models					
	DT	RF	SVM	LR	GBM	XGB
	Mean ± SD	Mean ± SD	Mean ± SD	Mean ± SD	Mean ± SD	Mean ± SD
demo	0.647 ± 0.093	0.672 ± 0.084	0.658 ± 0.091	0.722 ± 0.073	0.655 ± 0.093	0.679 ± 0.086
A	0.698 ± 0.094	0.788 ± 0.072	0.762 ± 0.080	0.756 ± 0.078	0.757 ± 0.076	0.792 ± 0.066
N	0.658 ± 0.091	0.763 ± 0.080	0.737 ± 0.066	0.648 ± 0.071	0.782 ± 0.080	0.766 ± 0.084
V	0.471 ± 0.096	0.495 ± 0.084	0.489 ± 0.088	0.558 ± 0.094	0.479 ± 0.079	0.467 ± 0.083
demo + A	0.722 ± 0.094	0.813 ± 0.073	0.788 ± 0.071	0.796 ± 0.071	0.803 ± 0.070	0.826 ± 0.063
demo + N	0.661 ± 0.093	0.782 ± 0.081	0.759 ± 0.063	0.644 ± 0.072	0.799 ± 0.071	0.780 ± 0.077
demo + V	0.610 ± 0.107	0.619 ± 0.086	0.646 ± 0.099	0.705 ± 0.091	0.599 ± 0.083	0.619 ± 0.084
demo + AN	0.722 ± 0.091	0.844 ± 0.063	0.826 ± 0.056	0.796 ± 0.064	0.881 ± 0.053	0.865 ± 0.057
demo + NV	0.667 ± 0.101	0.781 ± 0.080	0.758 ± 0.067	0.647 ± 0.073	0.798 ± 0.075	0.779 ± 0.083
demo + AV	0.716 ± 0.091	0.807 ± 0.072	0.778 ± 0.070	0.809 ± 0.067	0.803 ± 0.068	0.821 ± 0.061
demo + ANV	0.728 ± 0.098	0.840 ± 0.063	0.816 ± 0.055	0.799 ± 0.065	0.879 ± 0.053	0.863 ± 0.064

AUC; Area Under ROC Curve, SD; Standard Deviation, CI; Confidence Interval, DT; Decision Trees, RF; Random Forests, SVM; Support Vector Machines, LR; Linear Regression Classifiers, GBM; Gradient Boosting Models, XGB; Extreme Gradient Boosting, demo; demographic characteristics, A; amyloid PET image features, N; T1-weigted image features, V; T2-FLAIR image features.

### Selection of modality combinations

**Figure 1:**
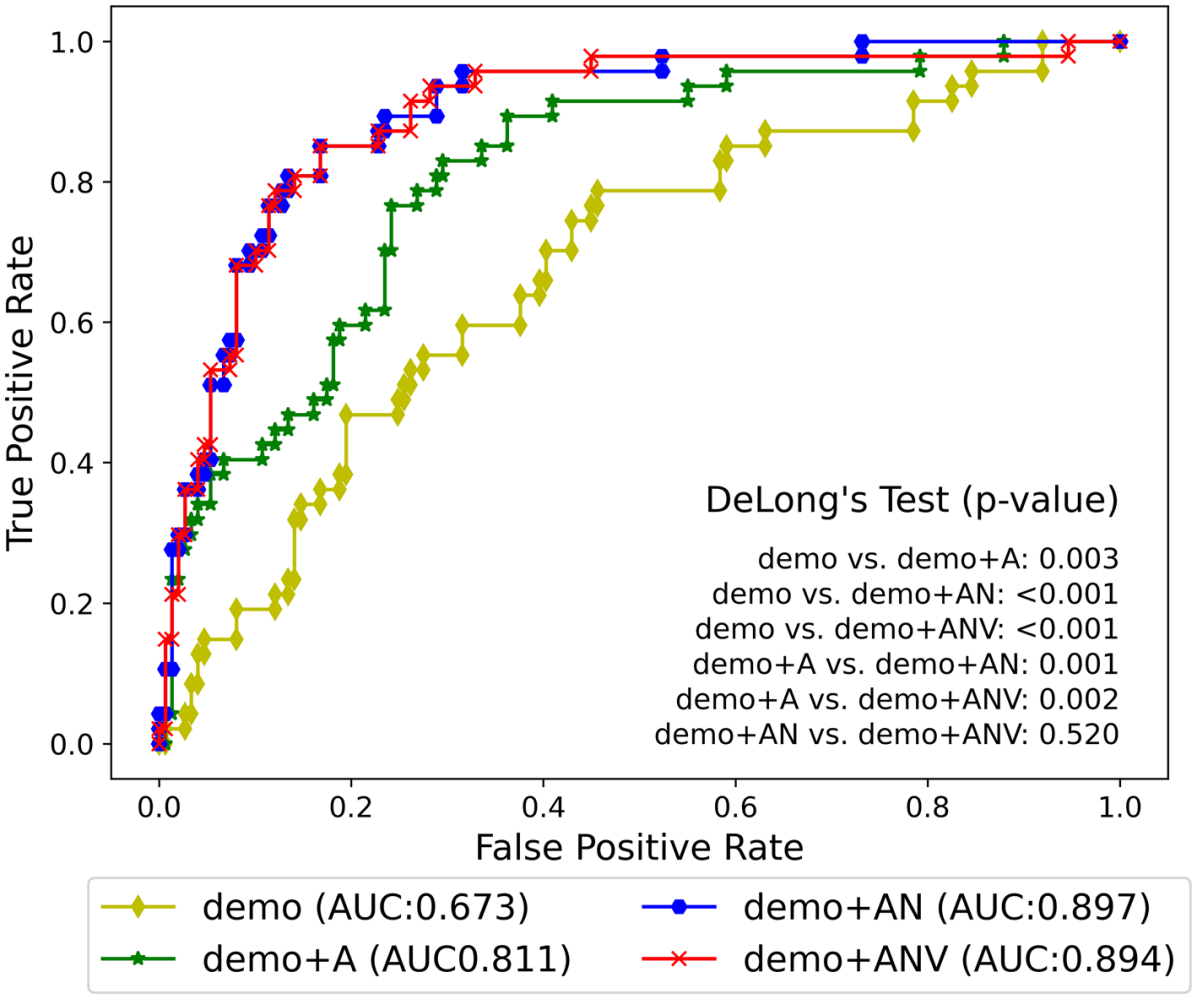
Performance comparison in AUC of GBM model for modality combinations of each testing set obtained by performing 100 iterations of data shuffling. AUC showed a statistically significant improvement in the modality combination that added image features compared to when only demographic characteristics were used (*p*-value < 0.05). There was a statistically significant improvement in the AUC when adding MRI image features to the model compared to using the demo + A modality combination (*p*-value < 0.05). However, there was no statistically significant difference in the AUC between the model using the demo + AN modality combination and the model using the demo + ANV modality combination (*p*-value = 0.520). GBM;gradient boosting model, demo; demographic characteristic, A;amyloid PET image features, N; T1-weighted image features, V; T2-FLAIR image features.

Among the modality combinations of the GBM model who’s the highest robustness was achieved in the model selection, the top three combinations with high AUC and the base modality combination (demo) were selected and statistical tests were performed. The selected combinations were demo+A, demo+AN, and demo+ANV. We investigated whether image features contribute to AD conversion prediction by performing a DeLong test as shown in Fig. 1. The performance of model using demo was statistically inferior to the model using demo+A (*p*=0.008), demo+AN (*p*<0.001), and demo+ANV (*p*<0.001) as shown in Fig. 1. The performance of demo+A were also statistically lower than one of demo+AN (*p*=0.001) and demo+ANV (*p*=0.002). The performance between demo+AN and demo+ANV were not different statistically (*p*=0.520).

#### Performance estimates of final model

**Figure 2:**
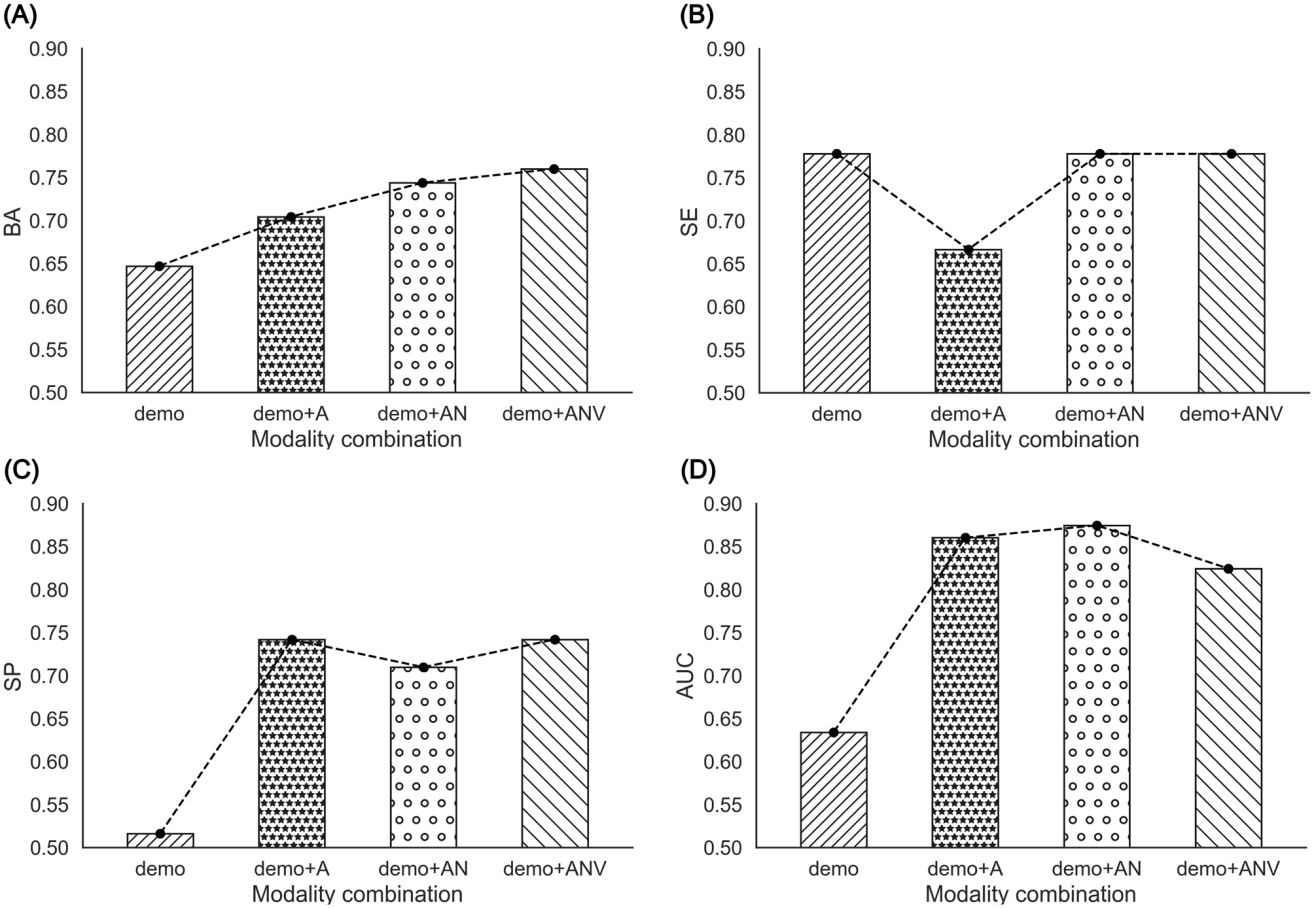
Performance comparison results of GBM models according to primary modality combination. (**A**) The modality combination with the highest BA is demo + ANV, 0.760. (**B**) The modality combination with the highest SE is demo, demo + AN, demo + ANV, 0.778. (**C**) The modality combination with the highest SP is demo + A, 0.742, (**D**) The modality combination with the highest AUC is demo + AN, 0.875. GBM;gradient boosting model, demo; demographic characteristic, A;amyloid PET image features, N; T1-weighted image features, V; T2-FLAIR image features.

After the prior test, the selected GBM model was used to estimate the performance of the final model using a fixed training set and testing set, and the modality combinations investigated were as follows: demo, demo+A, demo+AN, and demo+ANV. Table 4 describes the 10-fold cross-validation results for each modality combination. The results of testing set input to each tuned GBM model are shown in Fig. 2. The GBM model that predicted AD conversion using demographic characteristics showed a BA of 0.647, SE of 0.778, SP of 0.516, and AUC of 0.634. The AD conversion performance of the GBM model using demo+A modality combination showed a BA of 0.704, SE of 0.667, SP of 0.742, and AUC of 0.860. The AD conversion performance of the GBM model using demo+AN modality combination showed BA of 0.744, SE of 0.778, SP of 0.710, and AUC of 0.875. The AD conversion performance of the GBM model using demo+ANV modality combination showed BA of 0.760, SE of 0.778, SP of 0.742, and AUC of 0.824. As image features were added, BA gradually increased, but SE and SP were sometimes lower than the GBM model that used only demographic characteristics. The GBM model using the demo+AN modality combination showed the highest AUC, like the preliminary tests. However, unlike the AUC of the demo+AN modality combination and the demo+ANV modality combination, which showed no significant difference in Fig. 1, this result investigated that it was lower than the demo+A modality combination.

**Table 4:** Cross-validated GBM performance measures according to the modality combination.

Modality combination	Metrics			
	BA	SE	SP	AUC
	Mean ± SD	Mean ± SD	Mean ± SD	Mean ± SD
demo	0.768 ± 0.109	0.814 ± 0.170	0.721 ± 0.086	0.830 ± 0.079
demo + A	0.878 ± 0.075	0.925 ± 0.061	0.831 ± 0.147	0.937 ± 0.065
demo + AN	0.919 ± 0.048	0.949 ± 0.059	0.889 ± 0.084	0.977 ± 0.026
demo + ANV	0.923 ± 0.053	0.966 ± 0.044	0.881 ± 0.094	0.984 ± 0.016

GBM; Gradient Boosting Models. BA; Balanced Accuracy, SE; Sensitivity, SP; Specificity, AUC; Area Under ROC Curve, demo; demographic characteristics, A; amyloid PET image features, N; T1-weigted image features, V; T2-FLAIR image features.

## Discussion

This study aimed to investigate and validate a universally applicable machine learning model for predicting AD conversion in patients within a 4-year timeframe. By combining T1-weighted image features, T2-FLAIR image features, and amyloid PET image features based on demographic characteristics, we explored machine learning model selection and modality combinations with relatively good performance as part of a preliminary test. To overcome the challenge from our small dataset, we conducted model selection and selection of modality combination by performing data shuffling. Our model selection strategy was to perform 100 iterations of randomly generating training and testing sets^33^, and then select a model with good average performance of the trained model and low standard deviation. Furthermore, after model selection, we chose the top 3 modality combinations for the selected GBM model. We compared the AUC of the GBM model trained using demographics with the AUC of the GBM model trained using the selected modality combinations. Although there have been various studies attempting to predict AD conversion in MCI patients using machine learning, the strength of this article lies in verifying the robustness of the model and investigating the best modality combination from a small dataset. Through this process, we were able to establish experimental evidence for estimating the performance of final model using the pre-defined fixed dataset.

Previous studies have explored various modalities and techniques for predicting AD conversion in patients with MCI. Hinrichs, Chris, et al., utilized longitudinal MRI data in predicting AD conversion in patients with progressive MCI (MCIp)^34^. Moradi, Elaheh, et al., developed a prediction model in 1-3 years intervals^8^. They performed aggregation with MRI features and MMSE scores adjusted for age. Zhang, D. & Shen, D., et al., combined fluorodeoxyglucose (FDG)—PET, MRI, and cognitive scores^35^. Zhang, T. et al., proposed a framework using a combination of structural and functional MRI features^36^. Franciotti, Raffaella, et al., constructed a multi-modal dataset using neurophysiological test scores, cerebrospinal fluid (CSF), the ApoE genotype, and structural MRI features^37^. Lin, Weiming, et al., proposed a framework for developing a predictive model within three years using structural MRI features, FDG-PET, CSF, ApoE genotype, and neuropsychological scores. However, these studies included biomarkers thorugh invasive methods or used indicators obtained through neuropsychological tests, which take a long time to obtain, as factors^38^. In contrast to those studies, our focus was not only to investigate the predictability of AD conversion in MCI patients by combining the features of T1-weighted images, T2-FLAIR images, and *α*PET images, which are mainly used in clinical environment, but also to determine the modality combinations showing good performance. Our results showed that the AUC increased in a machine learning model that combined demographic characteristics with regional SUVR of *α*PET images and regional volume and HOC of T1-weighted images. These results have a similar context to previous studies that attempted to predict AD conversion using regional volume or regional SUVR [8–14,21,22]. However, the WMH information and Fazekas scale information of the T2-FLAIR image were not meaningful information in predicting AD conversion. These findings suggest that it is difficult to contribute to improving the performance of the AD conversion prediction model in MCI patients with only fragmentary information on the ratio of WMH volume compared to the white matter and the Fazekas scale of the T2-FLAIR image. Considering that there are reports that an increase in WMH is associated with a decline in cognitive function^17,39,40^, it was obvious that the WMH ratio used in this study at any time point did not contribute to the prediction of AD conversion. Therefore, if the feature of the T2-FLAIR image is used as information about the amount of change in WMH from longitudinal T2-FLAIR image, it is believed that the T2-FLAIR image features could also be placed in an important factor in the multi-modality combination. SUVR–14,21,22]. However, the WMH information and Fazekas scale information of the T2-FLAIR image were not meaningful information in predicting AD conversion. These findings suggest that it is difficult to contribute to improving the performance of the AD conversion prediction model in MCI patients with only fragmentary information on the ratio of WMH volume compared to the white matter and the Fazekas scale of the T2-FLAIR image. Considering that there are reports that an increase in WMH is associated with a decline in cognitive function^17,39,40^, it was obvious that the WMH ratio used in this study at any time point did not contribute to the prediction of AD conversion. Therefore, if the feature of the T2-FLAIR image is used as information about the amount of change in WMH from longitudinal T2-FLAIR image, it is believed that the T2-FLAIR image features could also be placed in an important factor in the multi-modality combination.

Although our study yielded promising results, some limitations must be acknowledged. First, we did not consider lifestyle patterns of patients with MCI, such as alcohol consumption, smoking, and exercise, which could potentially enhance the performance of our decision-making model. Incorporating this information into future studies may lead to better predictive values. Second, the best modality combination used in this study commonly used *α*PET image features. Although the *α*PET image features were helpful in further improving the model’s performance, the cost of *α*PET imaging and the radiation hazards of *α*PET still exist. Lastly, because the amount of data in the conversion group was small, the ratio of the non-conversion group and the conversion group was adjusted using the SMOTE technique. If the amount of data in the conversion group can be increased, it is believed that a suitable machine learning model can be found to explain the prediction of AD conversion in MCI patients through data shuffle. In this study, we observed how the results of the test set vary according to the modality combination in the machine learning model for predicting the conversion from MCI to AD. Due to the limited amount of data, we conducted experiments by shuffling the training set and test set to find the most robust model. Through this process, we developed a reliable model and evaluated the performance of the model for each modality combination. As a result, it was found that the probability of an MCI patient converting to AD within 2–4 years could be predicted through machine learning based on the individual’s demographic characteristics, regional volumes, HOC, and regional SUVRs. Our research results are expected to provide useful information to clinician in predicting the risk of conversion from MCI to AD, thereby influencing early diagnosis and the establishment of personalized treatment plans. By developing a reliable model and identifying the optimal modality combination, clinicians can perform more accurate and effective predictions.

### Supplementary Information

Supplementary Tables.

## Data Availability

The datasets used and analyzed during the current study available from the corresponding author on reasonable request.
