# Supplementary material for: A multimodal machine learning model for predicting dementia conversion in Alzheimer’s disease
Source: Sci Rep. 2024 May 29;14:12276. doi: 10.1038/s41598-024-60134-2 (PMC11133319; doi:10.1038/s41598-024-60134-2)
Supplement: Supplementary file 1 — Supplementary Tables. [file 41598_2024_60134_MOESM1_ESM.docx]

**Supplement Table 1.** Baseline characteristics of each site

|  |  | ADNI | Site1 | Site2 | Site3 | Site4 |
| --- | --- | --- | --- | --- | --- | --- |
| Non-converter | N | 40 | 44 | 2 | 34 | 29 |
|  | Age (years) | 71.52$\pm$5.46 | 75.09$\pm$5.49 | 76.00$\pm$3.00 | 71.82$\pm$6.96 | 65.90$\pm$8.19 |
|  | MMSE | 28.12$\pm$2.10 | 25.57$\pm$3.07 | 25.00$\pm$2.00 | 26.65$\pm$2.82 | 25.41$\pm$3.59 |
|  | Sex, % (N) | 40 (16) | 66 (29) | 50 (1) | 74 (25) | 48 (14) |
|  | CDR  (Baseline) | 0.5 | 0.5 | 0.5 | 0.5 | 0.5 |
|  | CDR  (Follow up) | 0.5 | 0.5 | 0.5 | 0.5 | 0.5 |
|  | ApoE ε4 Carrier, % (N) | 38 (15) | 36 (16) | 0 | 18 (6) | 31 (9) |
|  | Study interval  (years) | 2.69$\pm$0.50 | 2.65$\pm$0.51 | 2.23$\pm$0.18 | 2.41$\pm$0.37 | 2.71$\pm$0.57 |
| Converter | N | 12 | 18 |  | 3 | 14 |
|  | Age (years) | 72.87$\pm$6.51 | 74.61$\pm$5.75 |  | 70.33$\pm$4.50 | 71.43$\pm$7.60 |
|  | MMSE | 26.42$\pm$2.18 | 23.39$\pm$2.54 |  | 19.33$\pm$4.64 | 23.71$\pm$2.81 |
|  | Sex, % (N) | 50 (6) | 61 (11) |  | 67 (2) | 50 (7) |
|  | CDR  (Baseline) | 0.5 | 0.5 |  | 0.5 | 0.5 |
|  | CDR  (Follow up) | 1 | 1.22$\pm$0.42 |  | 1.33$\pm$0.47 | 1.07$\pm$0.26 |
|  | ApoE ε4 Carrier, % (N) | 50 (6) | 44 (8) |  | 100 (3) | 43 (6) |
|  | Study interval  (years) | 2.48$\pm$0.43 | 2.69$\pm$0.49 |  | 2.61$\pm$0.41 | 2.91$\pm$0.63 |

ADNI, Alzheimer’s Disease Neuroimaging Initiative; MMSE, Mini-mental State Examination; CDR, Clinical Dementia Rating; ApoE4, apolipoprotein E4. Chi-square tests for sex and ApoE genotype, two-sample t-tests for age and MMSE.

**Supplement Table 2.** Summary of imaging system and acquisition difference across each site

ADNI, Alzheimer’s Disease Neuroimaging Initiative; TR, Time Repetition; TI, Inversion Time; TE, Echo Time; FA, Flip Angle.

| **Modality** |  | **ADNI** | **Site1** | **Site2** | **Site3** | **Site4** |
| --- | --- | --- | --- | --- | --- | --- |
| T1 | 3T Scanner | GE  Philips  Siemens | Siemens | Siemens  GE | GE  Philips | Philips  Siemens |
|  | TR (ms) | 7.3 ~ 7.6  6.5  2300 | 1700~1800 | 2000  7.956 | 7.1 ~ 8.88  9.8 | 9.4  2000 |
|  | TE (ms) | 3.05 ~ 3.12  2.9  2.95 ~ 2.98 | 2.6 | 2.29  2.82 | 2.776~3.396  4.6 | 4.6  3.05 |
|  | FA | 9$^{\circ}$ or 11$^{\circ}$ | 9$^{\circ}$ | 8$^{\circ}$ or 10$^{\circ}$ | 8$^{\circ}$ or 12$^{\circ}$ | 8$^{\circ}$ or 9$^{\circ}$ |
| T2 FLAIR | TR/TI (ms/ms) | 4800/1442~1482  4800/1650  4800 or 9000/1650~2500 | 9000/2500 | 9000/2500  11000/2648.61 | 8800~12000/2450~2709  8000/2500 | 10000/2800  8000~10730/2500~2665 |
|  | TE (ms) | 115.7~117  271~275  90~443 | 76 | 121  93.544 | 89~128  125 | 120 or 125  86~115 |
|  | FA | 90$^{\circ}$ or 120$^{\circ}$ | 150$^{\circ}$ | 121$^{\circ}$ or 160$^{\circ}$ | 90$^{\circ}$ or 160$^{\circ}$ | 90$^{\circ}$ or 150$^{\circ}$ |
| $\alpha$PET | Ligand | ^18^F-Florbetapir  ^18^F-Florbetaben | ^18^F-Florbetaben  ^18^F-Flutemetamol | ^18^F-Flutemetamol | ^18^F-Flutemetamol | ^18^F-Florbetaben |
